# Supplementary material for: Impaired Heat Shock Protein Expression in Activated T Cells in B-Cell Lymphoma
Source: Biomedicines. 2022 Oct 28;10(11):2747. doi: 10.3390/biomedicines10112747 (PMC9687880; doi:10.3390/biomedicines10112747)
Supplement: Supplementary file 1 [file biomedicines-10-02747-s001.zip › biomedicines-1957697-supplementary.pdf]

Supplementary materials

Supplementary Figure

A **B**

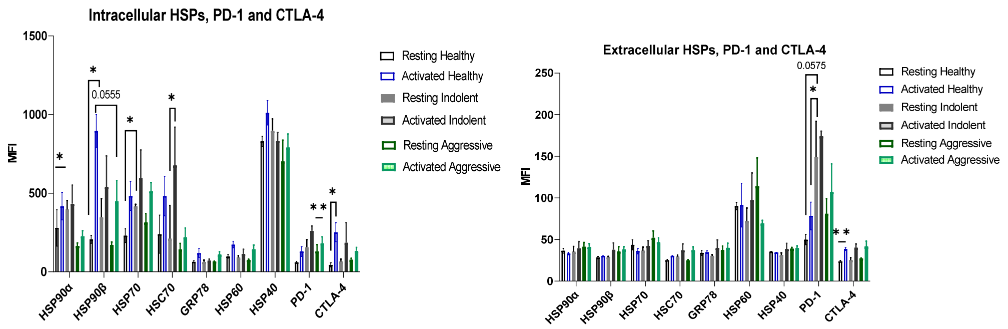

Figure S1. HSP, PD-1 and CTLA-4 expression in activated T cells. Intracellular (**A**) and extracellular (**B**) expression of HSPs, PD-1, CTLA-4 in resting and activated T cells in healthy controls and lymphoma patients.
